# *TFAP2B* Haploinsufficiency Impacts Gastrointestinal Function and Leads to Pediatric Intestinal Pseudo-obstruction

**DOI:** 10.3389/fcell.2022.901824

**Published:** 2022-07-08

**Authors:** Almira Zada, Laura E. Kuil, Bianca M. de Graaf, Naomi Kakiailatu, Jonathan D. Windster, Alice S. Brooks, Marjon van Slegtenhorst, Barbara de Koning, René M. H. Wijnen, Veerle Melotte, Robert M. W. Hofstra, Erwin Brosens, Maria M. Alves

**Affiliations:** ^1^ Department of Clinical Genetics, Erasmus Medical Centre-Sophia Children’s Hospital, Rotterdam, Netherlands; ^2^ Department of Pediatric Surgery, Erasmus Medical Centre-Sophia Children’s Hospital, Rotterdam, Netherlands; ^3^ Department of Pediatric Gastroenterology, Erasmus University Medical Centre-Sophia Children’s Hospital, Rotterdam, Netherlands; ^4^ Department of Pathology, GROW-School for Oncology and Developmental Biology, Maastricht University Medical Center, Maastricht, Netherlands

**Keywords:** chronic intestinal pseudo-obstruction, enteric nervous system, intestinal motility, crispant, ednrbb

## Abstract

**Background:** Pediatric Intestinal Pseudo-obstruction (PIPO) is a congenital enteric disorder characterized by severe gastrointestinal (GI) dysmotility, without mechanical obstruction. Although several genes have been described to cause this disease, most patients do not receive a genetic diagnosis. Here, we aim to identify the genetic cause of PIPO in a patient diagnosed with severe intestinal dysmotility shortly after birth.

**Methods:** Whole exome sequencing (WES) was performed in the patient and unaffected parents, in a diagnostic setting. After identification of the potential disease-causing variant, its functional consequences were determined *in vitro* and *in vivo*. For this, expression constructs with and without the causing variant, were overexpressed in HEK293 cells. To investigate the role of the candidate gene in GI development and function, a zebrafish model was generated where its expression was disrupted using CRISPR/Cas9 editing.

**Results:** WES analysis identified a *de novo* heterozygous deletion in *TFAP2B* (NM_003221.4:c.602-5_606delTCTAGTTCCA), classified as a variant of unknown significance. *In vitro* studies showed that this deletion affects RNA splicing and results in loss of exon 4, leading to the appearance of a premature stop codon and absence of TFAP2B protein. Disruption of *tfap2b* in zebrafish led to decreased enteric neuronal numbers and delayed transit time. However, no defects in neuronal differentiation were detected. *tfap2b* crispants also showed decreased levels of *ednrbb* mRNA, a downstream target of *tfap2b*.

**Conclusion:** We showed that *TFAP2B* haploinsufficiency leads to reduced neuronal numbers and GI dysmotility, suggesting for the first time, that this gene is involved in PIPO pathogenesis.

## Introduction

Chronic intestinal pseudo-obstruction (CIPO) is characterized by severe intestinal dysmotility that imitates mechanical obstruction, and mainly affects the small bowel and colon (Di Nardo et al., 2017). CIPO represents a spectrum of heterogenous disorders with multiple pathological mechanisms, affecting the structure and/or function of the intestinal neuromuscular and interstitial cells of Cajal (Knowles et al., 2010; Downes et al., 2018). CIPO has been recognized in adult and children, where its etiology, onset, clinical features, and natural history harbor a fundamental difference. Therefore, when diagnosed in children, CIPO is considered a separate entity and referred to as pediatric intestinal pseudo-obstruction (PIPO) (Thapar et al., 2018). Overlapping histopathological features with other gastrointestinal neuromuscular disorders, lack of molecular biomarkers and no single diagnostic test which is pathognomonic for CIPO/PIPO, hinder this disorder to be early diagnosed (Downes et al., 2018). Consequently, most cases remain undiagnosed until advanced stages of disease. In addition, only few epidemiological studies reporting CIPO prevalence in the pediatric population have been documented (Muto et al., 2014), thus the prevalence of PIPO worldwide is difficult to estimate. From a genetic perspective, the majority of PIPO cases are sporadic with a *de novo* dominant pattern of inheritance (Gamboa and Sood, 2019). Mutations in *FLNA* following an X-linked pattern of inheritance (Gargiulo et al., 2007; Van Der Werf et al., 2013; Jenkins et al., 2018) and autosomal dominant mutations in *ACTG2* (Halim et al., 2016; Milunsky et al., 2017; Ravenscroft et al., 2018) have been identified in PIPO patients. In addition, mutations in *TYMP* (Nishino et al., 1999), *POLG1* (Van Goethem et al., 2003; Giordano et al., 2009), *SGOL1* (Chetaille et al., 2014) and *RAD21* (Bonora et al., 2015), which follow an autosomal recessive inheritance pattern, have also been linked to this disease. Despite the identification of several genes, for the majority of PIPO patients, the underlying genetic cause and molecular mechanisms remain unknown.

Here, we report for the first time, a PIPO patient carrying a *de novo* heterozygous deletion in the Transcription Factor Activating Protein-2 Beta (*TFAP2B*) gene (NM_003221.4:c.602-5_606delTCTAGTTCCA). *TFAP2B* encodes for a member of the AP-2 transcription factors family, which in mammals include TFAP2A, TFAP2B, TFAP2C, TFAP2D and TFAP2E (Hilger-Eversheim et al., 2000). TFAP2B is highly expressed in migrating neural crest cells (NCCs) during embryonic development of vertebrates (Hong et al., 2008; Hong et al., 2011; Schmidt et al., 2011). Since NCCs give rise to various tissues including peripheral neurons and glia, cardiovascular tissue, melanocytes, craniofacial bone and teeth, cartilage, and connective tissue (Prasad et al., 2019), variants in *TFAP2B* have been associated with different disorders, often classified as neurocristopathies. Char syndrome (OMIM#169100), characterized by patent ductus arteriosus (PDA), facial dysmorphism and hand anomalies, was the first known congenital genetic disorder linked to *TFAP2B*. This syndrome is caused by heterozygous missense variants in the gene, suspected to result in a dominant negative effect (Satoda et al., 2000; Zhao et al., 2001; Edward et al., 2019). The same type of variants have also been reported in patients with severe dental anomalies and incomplete penetrance of PDA (Tanasubsinn et al., 2017)*,* as well as in a pediatric Char patient diagnosed with central diabetes insipidus, scoliosis and hearing loss (Edward et al., 2019). Moreover, *TFAP2B* loss of function variants have been linked to Char syndrome with variable expressivity of PDA (Mani et al., 2005), familial and non-familial isolated PDA (Khetyar et al., 2008; Chen et al., 2011; Xiong et al., 2013; Ji et al., 2014) and syndromic craniosynostosis (Timberlake et al., 2019). Genome wide association studies and animal models have also shown an involvement of *TFAP2B* in metabolic syndromes and obesity risk (Kraja et al., 2014), as well as retinal (Jin et al., 2015) and renal development (Moser et al., 2003). However, this gene has never been involved in GI complaints, despite being recently described as one of the regulators of the Endothelin Receptor Type B (*EDNRB*) expression (Ling and Sauka-Spengler, 2019), a known Hirschsprung disease (HSCR) gene involved in GI development and function (Bondurand et al., 2018).

## Material and Methods

### Patient Characteristics

The first child of non-consanguineous Dutch parents, a boy, presented shortly after birth with biliary vomiting. One day post-partum, an abdominal X-ray showed distended bowel loops without air in the rectum. A contrast study was performed and showed slow passage from stomach to duodenum. A laparotomy also showed distended small bowel loops, which gradually tapered to a small lumen. Full-thickness biopsies were taken on different sites of the intestine, which showed normal ganglion cells, ruling out HSCR. Since this procedure, spontaneous passage of feces was hardly seen. At a few months of age, an antro-duodenal manometry study was performed overnight, showing abnormal small bowel motor activity, suggestive of a neuropathic phenotype. Unfortunately, colonic manometry was aborted due to a small caliber colon and risk of bleeding and perforation. A venting gastrotomy was performed to discharge gastric fluid. Currently, the patient is still alive, but total parenteral nutrition is given to support feeding. Informed consent was obtained from the parents for diagnostic genetic analysis.

### DNA Isolation, Whole Exome Sequencing Analysis and Variant Interpretation

DNA isolation from peripheral blood of patient and parents was performed using the Chemagic DNA Blood 4 k Kit (PerkinElmer, Waltham, MA, United States). Three micrograms of double-stranded DNA were fragmented (Covaris, Woburn, MA, United States) and exonic sequences were captured using the Agilent SureSelect Clinical Research Exome V2 (Agilent, Santa Clara, CA, United States). Paired-end sequencing was performed on a HiSeq 4,000 platform (150bp paired end) and an average coverage of at least 50X. Reads were mapped against the human reference genome GRCh37/hg19 with the Burrows-Wheeler Aligner (Li and Durbin, 2009), and variants were called using the Genome Analysis toolkit (Broad Institute, Cambridge, MA, United States). Alissa Interpret software (Agilent, Santa Clara, CA, United States) was used to filter and prioritize variants. Variants were classified according to the American College of Medical Genetics and Genomics (ACMG) standards and guidelines for the interpretation of sequence variants (Richards et al., 2015).

### Sanger Sequencing of *TFAP2B*

For validation of the *de novo* deletion identified in *TFAP2B*, Sanger sequencing was performed as previously described (Sribudiani et al., 2018), using the following primers: 5′ CCT​GGT​TCC​CAG​CAC​AGT​CC 3’ (forward) and 5′ CATTCAGGGGGCGACAGC 3’ (reverse). Touchdown 65°C–55°C PCR program was used to amplify target genomic region. PCR products underwent ExoSAP treatment and BDT reaction, and were sequenced on both strands using the BigDye v3.1 kit (Life Technologies, Carlsbad, CA, United States). Electropherograms were visualized with Chromas Lite v 2.1 (www.technelysium.com.au).

### *TFAP2B* Minigene for Exon Trapping Assay

An *in vitro* splicing assay was carried out using a pSPL3 exon-trapping vector (Addgene, Watertown, MA, United States). Briefly, the pSPL3 vector contains a small artificial gene composed of a SV40 promoter and exon splice donor vector (SD)-intron-exon splice acceptor vector (SA) sequence with functional splice donor and acceptor sites. A late polyadenylation signal is also present. Genomic DNA fragment from control and patient that comprises entire *TFAP2B* exon 4 plus additional 233 basepairs (bp) (5′) and 309 bp (3′) of the flanking intronic region was amplified by PCR, with primers containing additional *Xho*I (forward) and *BamH*I (reverse) restriction sites. The following primers: 5′ CAT​ATA​CTC​GAG​CCT​GGT​TCC​CAG​CAC​AGT​CC 3’ (forward) and 5′ TAT​CGT​GGA​TCC​CAT​TCA​GGG​GGG​CGA​CAG​C 3’ (reverse) were used for this purpose. After PCR amplification, products were purified and subjected to restriction enzyme digestion. They were subsequently inserted into the pSPL3 vector to create a minigene construct. The minigene constructs were Sanger sequenced to confirm the presence of the wild type and mutant DNA fragments.

### Expression Vectors

pCMV-Myc-tagged AP2 beta (*TFAP2B*) was purchased from Origene (OriGene, Rockville, Maryland, United States). The following *TFAP2B* variants: deletion of whole exon 4, c.706 C > T, c.898 C > T and c.1144 C > T were generated by site directed mutagenesis according to the Q5 Site Directed Mutagenesis manufacturer’s instructions (New England Biolabs, Ipswich, MA, United States). Primers used in site directed mutagenesis experiment were listed in Supplementary Table S1. Following mutagenesis, the entire *TFAP2B* insert was evaluated by Sanger sequencing, using the following primers: 5′ GCA​TGG​GTG​ACA​GCC​TCT​CG 3’ (forward) and 5′ GGT​CAC​TCG​GGT​CTG​TGT​C 3’ (reverse).

### Cell Culture and Transfection

Human Embryonic Kidney cells (HEK293) were cultured in DMEM (Lonza, Basel, Switzerland), supplemented with 10% fetal calf serum (Sigma-Aldrich, Burlington, MA, United States) and 1% penicillin/streptomycin (Gibco-Life Technologies, Renfrewshire, United Kingdom). Cells were maintained at 37°C and 5% CO_2_. For transient transfection, 500,000 cells were seeded in 6 well-plates. Twenty-four hours (h) after, cells were transfected with the wild type and mutant constructs, using GeneJuice Transfection Reagent (MilliporeSigma, Burlington, MA, United States), according to the manufacturer’s instructions. Experiments were performed in duplicate.

### RNA Isolation, cDNA Preparation and qRT-PCR

Total RNA was harvested 48 h post transfection using the RNeasy Mini Kit (Qiagen, Hilden, Germany). Total RNA was quantified using the NanoDrop (Thermo Fisher Scientific, Waltham, MA United States) and 1 ug RNA was reverse transcribed using the iScript™ cDNA Synthesis Kit (Bio-Rad, Hercules, CA, United States), according to the manufacture’s protocol. Gene expression levels of *TFAP2B* were measured by quantitative real time (qRT)-PCR using iTaq universal SYBR Green Supermix (Bio-Rad, Hercules, CA, United States). The following primers: 5′ TAT​GAG​GAC​CGG​CAC​GAT​G 3’ (forward) and 5′ GTAGGGCGGCTGGAAGTC 3’ (reverse) were used for amplifying the *TFAP2B* transcript. *GAPDH* (5′ CGA​CCT​TCA​CCT​TCC​CCA​T 3’ (forward) and 5′ TAA​AAG​CAG​CCC​TGG​TGA​CC 3’ (reverse))and *β-Actin* (5′ AAC​CGC​GAG​AAG​ATG​ACC​C 3’ (forward primer) and 5′ GCC​AGA​GGC​GTA​CAG​GGA​TAG 3’ (reverse primer)) were used as housekeeping genes. Two independent experiments were performed for statistical analysis.

### Cell Lysates and Western Blot Analysis

Protein lysates were collected 48 h post-transfection. Cells were washed with PBS and incubated with lysis buffer containing 150 mM NaCl, 20mMTris, 1% Triton X, 1x protease inhibitors cOmplete™ (Roche, Basel, Switzerland), for 30 min on ice. Lysates were stored at −80°C. Protein quantification was performed using the Pierce BCA kit (Thermo Fisher Scientific, Waltham, MA, United States) and 40 ug of protein was loaded into a criterion TGX precast gel (Bio-Rad, Hercules, CA, United States). The following primary antibodies were used: Myc 1:3,000 (Cell Signaling Technology, Danvers, MA, United States), *β*-Actin 1: 1,000 (Santa Cruz Biotechnology, Santa Cruz, CA, United States), and GFP 1:2,000 (AbCam, Cambridge, United Kingdom). Secondary antibodies used were IRDye 800CW Goat anti mouse (Li-Cor, Lincoln, NE, United States) and IRDye 680RD Goat anti-Rabbit (Li-Cor, Lincoln, NE, United States).

### Zebrafish Larvae Maintenance

Zebrafish Tg (*phox2bb*:GFP) embryos and larvae (Nechiporuk et al., 2007) were kept at 28°C on a 14–10 h light/dark cycle in 1 M HEPES buffered (pH 7.2) E3 medium (34.8 g NaCl, 1.6 g KCL, 5.8 g CaCl2.2H2O, 9.78 g MgCl2). For quantification of enteric neurons, E3+ 0,003% 1-phenyl 2-thiourea (PTU) was added to the medium at 1 day post fertilization (dpf) to prevent pigmentation. Animal experiments were approved by the Animal Experimentation Committee of the Erasmus MC, Rotterdam (No. AVD1010020209425).

### Zebrafish Crispant *tfap2b* Generation

*tfap2b* specific guide RNA (gRNA) was designed to target the beginning of exon 4 (5′ CGT​CAA​CGA​GGT​TTT​CTG​CT 3’, https://www.idtdna.com/). The synthesized gRNA (1 µL) was mixed with 4 ng of Cas9-nuclease and the final volume was adjusted to 6 µL with 300 mM KCl. Approximately, 1 nL of the mix was injected into an one-cell stage fertilized zebrafish oocytes. Genomic DNA surrounding the gRNA target site was Sanger sequenced and efficiency of insertions/deletions (InDels) was determined using the online tool TIDE (shinyapps.datacurators.ni/tide/), as previously described (Brinkman et al., 2014; Kuil et al., 2019). For neuronal quantification and intestinal transit assay, gRNA efficiency was evaluated for each individual larva. For genes transcript expression analysis, the gRNA efficiency was determined from a pool of 25 larvae. We refer to the generation of larvae that are directly injected with gRNA/Cas9 protein complex as crispants (F0). F0 fish were maintained until adulthood to generate stable mutants.

### Generation of *tfap2b* ^*+/−*^ Mutant Zebrafish Stable Line

Fin clips of founder (F0) fish were used for DNA isolation using a mixture containing Tris-HCl (pH9.0), KCl, Triton X-100, and protease K (Sigma Aldrich, St. Louis, Missouri, United States). After incubation at 55°C for 1 h, protease K was inactivated at 98°C for 10 min. The digested fin mx was used directly as a template for a PCR reaction using a standard PCR touchdown program (65°C–55°C) and the following primers: 5′ TCC​ACG​CAC​AGT​TCC​AGT​TCC 3’ (forward) and 5′ ACC​CCA​CCA​AGC​AGA​GAC​GC 3’ (reverse) to amplify the genomic target. Sanger sequencing was used to confirm the presence of InDels in *tfap2b*. Sanger reads were analyzed using Chromas Lite v 2.1 (www.technelysium.com.au). F0 were crossed out with wild-type fish to generate F1 fish. F1 fish with a heterozygous deletion of 7 bp in exon 4 of *tfap2b* (ENSDART00000174808.2: c.629-635delTTTTCTG, frame shift) were maintained (Supplementary Figures S1A,B). For further experiments, F1 fish were crossed, and the offspring was used to determine the number of enteric neurons and differentiated enteric neurons, as well as for intestinal transit time experiments.

### Quantification of Enteric Neuronal Numbers in Zebrafish

Five dpf F2 zebrafish larvae (phox2bb:GFP) treated with PTU were used to determine the number of enteric neurons in the gut. For imaging, larvae were anaesthetized with 0.016% Tricaine (MS-222) and placed on a 1.8% agarose coated Petridish, to be observed under the fluorescent microscope (Leica M165FC). Fish containing the transgene were selected using the GFP signal and images were taken. The enteric neurons were counted using ImageJ software (National Institute of Health, Maryland, United States). All larvae were genotyped at the end of the experiment. The number of enteric neurons were also analysed in F0 crispants, cas9 injected and uninjected embryos.

### Zebrafish Intestinal Transit Time Assay

To increase feeding efficiency, 5 dpf F2 larvae were fed for 1 day with dry food pellet. At 7 dpf, larvae (control group n = 30, crispant group = 29) were fed with a fluorescence pellet generated by mixing the dry food pellet with FluoSpheres carboxylate (Invitrogen, Waltham, MA, United States). After 2 h, fish with pellet located in zone 1 of the gut (0 h) were selected and placed in a new Petridish, at 28°C. After 16 h, fish were visualized again with the fluorescent microscope (Leica M165FC) to determine location of the pellet in the gut. All larvae were genotyped at the end of the experiment.

### Zebrafish RNA Isolation and Expression Analysis

Zebrafish F0 crispant and uninjected larvae were collected at six different time points during development: 8 h post fertilization (hpf), 1, 2, 3, 4, and 5 dpf, snapped frozen and stored at −80°C (n = 50 per time point). RNA from these larvae was isolated using Trizol reagent (Ambion, Austin, TX, United States) and 500 ng of total RNA was reverse transcribed using the iScript™ cDNA Synthesis Kit (Bio-Rad, Hercules, CA, United States), according to the manufacture’s protocol. *elfa* (5′ TTG​AGA​AGA​AAA​TCG​GTG​GTG​CTG 3’ (forward primer) and 5′ GGA​ACG​GTG​TGA​TTG​AGG​GAA​ATT​C 3’ (reverse primer)) and *β-actin* (5′ CGA​GCA​GGA​GAT​GGG​AAC​C 3’ (forward primer) and 5′ CAA​CGG​AAA​CGC​TCA​TTG​C 3’ (reverse primer)) were used as housekeeping genes. Comparison of the expression levels of crispants and wild type embryos was performed for each analyzed developmental stage. Two independent technical replications were performed for the statistical analysis.

### Quantification of the Proportion of Differentiated Enteric Neurons in Zebrafish

To determine the proportion of terminally differentiated enteric neurons, 5 dpf F2 zebrafish larvae (*tfap2b* ^*+/-*^, *phox2bb*:GFP) treated with PTU were immunostained with antiHuC/HuD, a mature neuronal marker. Larvae were incubated on ice for 30 min before being fixed in 4% PFA and washed in 1x phosphate buffer solution/0.25% Triton X-100 for 1 h, at room temperature. Whole mount antibody staining was performed according to previous reports (Uyttebroek et al., 2010). Anti HuC/HuD (1:100, A-21271, Invitrogen, Waltham, Massachusetts, United States) was used as primary antibody and Cy3 Mouse IgG (1:500, Thermo Fisher Scientific, Waltham, Massachusetts, United States) as secondary antibody. Larvae were imaged under the confocal microscope (Leica SP5 AOBS, Leica Camera, Wetzlar, Germany). The number of phox2bb:GFP^+^ and HuC/HuD^+^ cells were counted using Fiji ImageJ software. The proportion of differentiated enteric neurons was determined by calculating the ratio of HuC/HuD^+^ cells to phox2bb:GFP^+^ cells. All larvae were genotyped at the end of the experiment.

### Statistical Analysis

RNA expression results of HEK293-transfected cells and zebrafish were presented as fold change expressions. Potential differences between two groups were evaluated using an unpaired *t*-test (http://graphpad.com/). The number of enteric neurons present in the zebrafish gut is presented as the number of neurons per 100 µm and the enteric neuronal differentiation is presented as ratio of HuC/HuD^+^:phox2bb:GFP^+^. Potential difference between groups was tested using an unpaired *t*-test from the GraphPad package. Differences in zone location for the intestinal transit time assay were tested using the Proportion’s Test (https://www.medcalc.org/calc/comparison_of_proportions.php).

## Results

### Identification of a Novel *TFAP2B* Deletion in a PIPO Patient

**FIGURE 1:**
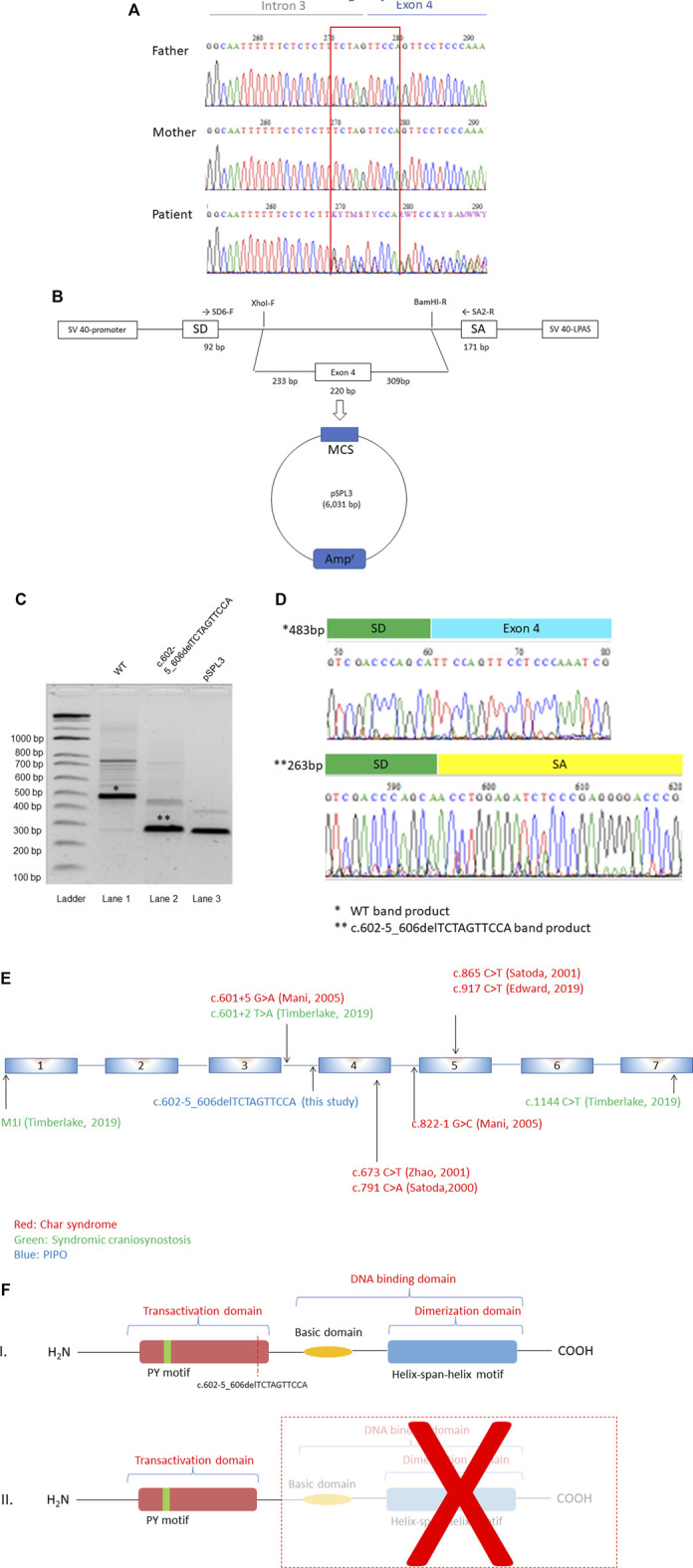
(Continued).

Whole exome sequencing (WES) was performed on genomic DNA isolated from proband and both parents. Data analysis identified a *de novo* heterozygous deletion in *TFAP2B* (NM_003221.4:c.602-5_606delTCTAGTTCCA, frame shift) which was located at the intron 3-exon 4 boundary (Figure 1A). This deletion was predicted to affect a canonical splice site and was therefore, considered ‘potentially deleterious’. However, since this was the first time a variant in this gene was identified in a PIPO patient, it was classified as a variant of unknown significance (VUS). This deletion was registered in ClinVar database with accession number SCV002507296. This exact deletion is absent from GnomAD (control cohort), but three loss of function variants in *TFAP2B* (c.82-1254C > A, MAF = 0.000006590; and c.602-2_602delTAG, MAF = 0.00001315, in European population; c.419C > A, MAF = 0.000006570, in African-American population) have been documented (GnomADv3.1.2). One of these variants (c.602-2_602delTAG) affects the same region deleted in our PIPO patient.

### *TFAP2B* Deletion Leads to Exon Skipping and the Appearance of a Premature Stop Codon

To test pathogenicity of the *TFAP2B* deletion, we first evaluated the potential effect of the c.602-5_606delTCTAGTTCCA variant, on splicing. Minigenes (Figure 1B) that carried the wild type and mutant sequences were transfected into HEK293 cells. Reverse transcriptase (RT)-PCR analysis was performed on total RNA isolated from transfected cells and detected a 483 bp band in cells transfected with the wild type plasmid (Figure 1C). Direct sequencing of this band revealed that it corresponded to exon 4, as expected. In contrast, the mutant minigene showed a shorter fragment which lacked exon 4 (Figure 1D). This deleted exon 4 is located in the transactivation domain of TFAP2 family protein (http://us.expasy.org/sprot/), together with other variants described in syndromic craniosysnostosis (Timberlake et al., 2019) and syndromic PDA (Mani et al., 2005) depicted in Figure 1E. Skipping of exon 4 leads to a premature stop codon at position c.863, and therefore we considered this deletion to be pathogenic (Figure 1F).

### Deletion of Exon 4 Leads to Reduced Expression Levels of *TFAP2B in vitro*

**FIGURE 2:**
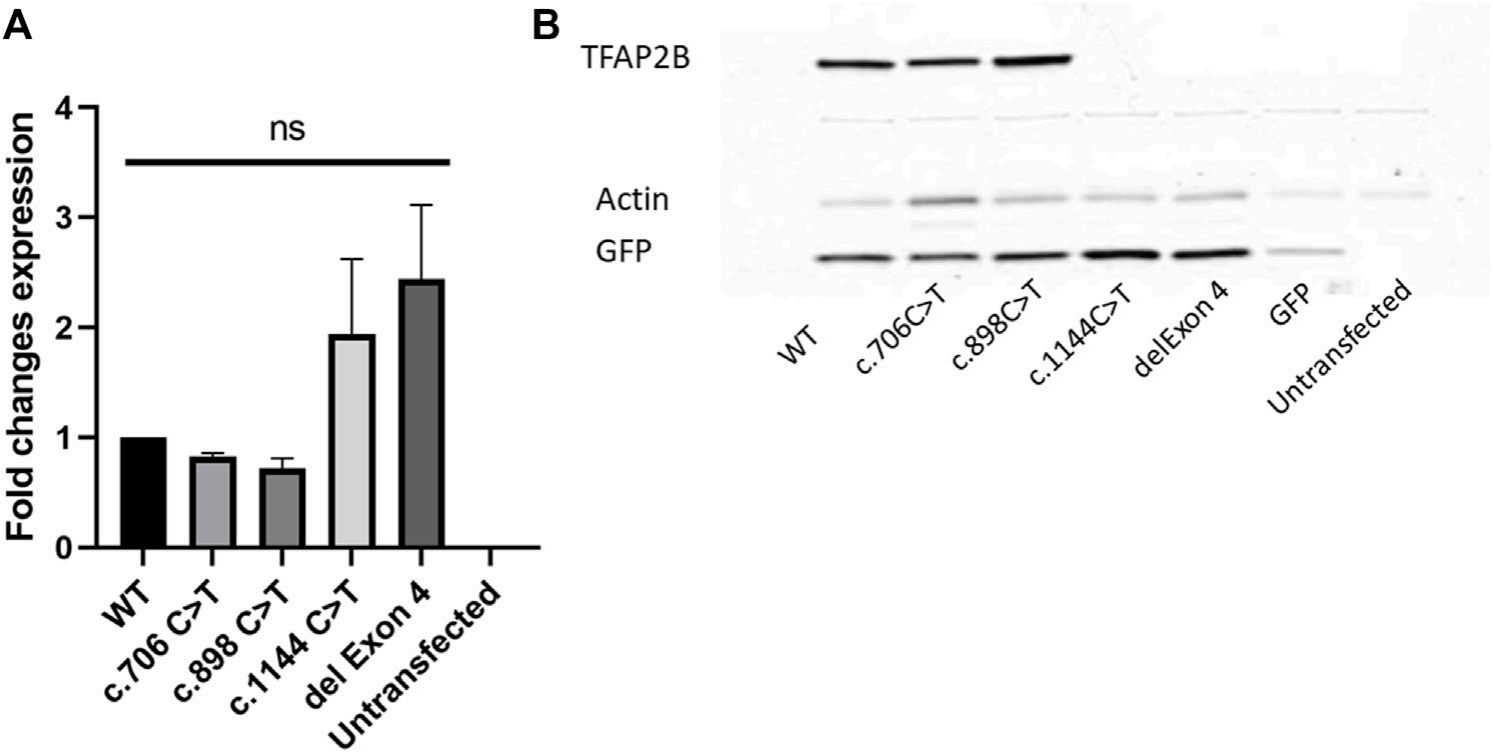
TFAP2B transcript and protein expression analysis. **(A)** qRT-PCR analysis of *TFAP2B* transcript levels of WT, c.706 C > T, c.898 C > T, c.1144 C > T, and exon 4 deletion expression constructs show no statistically significant difference among the samples normalized to *GAPDH* and *b-Actin*. **(B)** Western blot analysis of all tested variants shows absence of protein products for the syndromic craniosynostosis variant (c.1144 C > T) and PIPO variant (del Exon 4). Protein products are still detectable for the Char syndrome variants (c.706 C > T and c.898 C > T). A Myc-tag antibody was used to visualize TFAP2B protein levels.

To study the impact of the exon 4 deletion identified, we determined the expression levels of *TFAP2B* in HEK293 cells, by overexpressing a construct containing the wild type *TFAP2B* and the exon 4 deletion, tagged C-terminally with a Myc-tag. For comparison, we also included two other *TFAP2B* constructs overexpressing missense variants known to cause Char syndrome (c.706 C > T and c.898 C > T) and one construct containing a nonsense variant identified in craniosynostosis (c.1144 C > T). qRT-PCR analysis of *TFAP2B* showed a slight increase in mRNA levels for the variants associated with craniosynostosis and CIPO, while missense variants associated to Char syndrome showed a slight decrease of transcript levels when compared to the wild type (Figure 2A). However, neither of these changes was statistically significant. Subsequent protein analysis showed absence of TFAP2B protein for the constructs containing the deletion identified in the PIPO patient, as well as the nonsense variant identified in craniosynostosis. For both the Char syndrome variants, protein levels of TFAP2B were still detectable and comparable to the wild type (Figure 2B), which is in line with the suspected dominant negative effect previously reported for missense variants associated with this syndrome (Zhao et al.,2001).

### Loss of tfap2b Leads to Decrease Enteric Neuronal Numbers and Delayed Intestinal Transit Time in a Zebrafish Model

To determine if *TFAP2B* is involved in the development and function of the GI tract, we disrupted this gene in zebrafish using CRISPR/Cas9 editing. Zebrafish Tfap2b shares 88.71% sequence identity with the human protein (Supplementary Figure S1A). Based on the literature, *tfap2b* is only expressed in neural crest-derived cells in early embryonic development (Bradford et al., 2022) and in differentiating distal nephron segment at the 28 somite-stage (Chambers et al., 2019). At 5 dpf, *tfap2b* seems to be only expressed in the optic tectum, retinal inner layer, and spinal cord, with no expression expected in the intestine (Thisse and Thisse, 2004).To disrupt expression of tfap2b, the Cas9 protein/gRNA complexes were injected into fertilized zebrafish oocytes at one-cell stage. The gRNA was specifically designed to target the beginning of exon 4 of *tfap2b*. Efficiency of targeting was evaluated by the generation of InDels and was determined by Sanger sequencing of genomic DNA from individual 5dpf larvae (n = 48). The TIDE program was used to check sequence decomposition, showing that on average 73.46% (SD ± 11.288) of the alleles harbored InDels (i.e mutagenesis efficiency).

**FIGURE 3:**
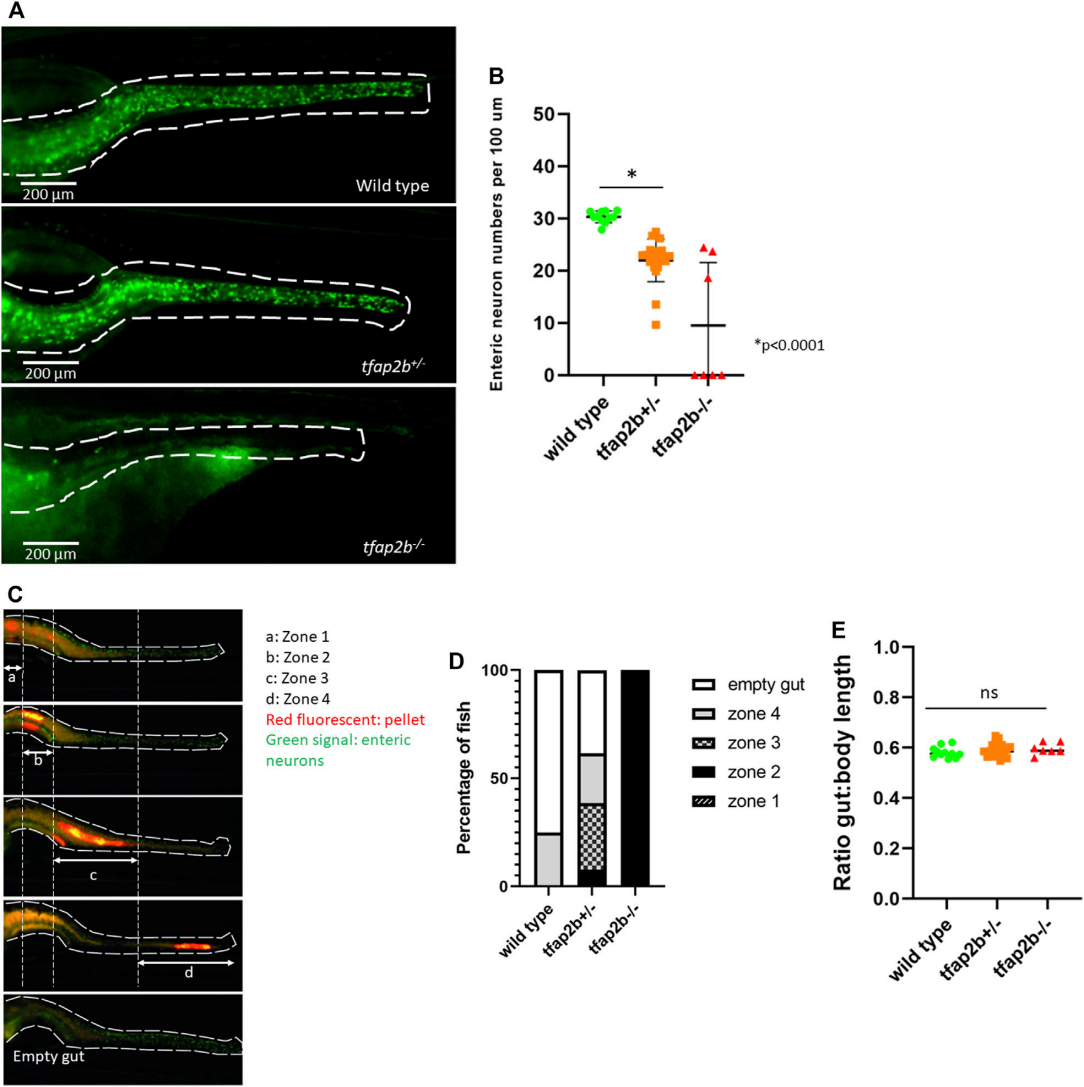
Decreased enteric neuronal numbers and delayed intestinal transit time in *tfap2b* ^*+/−*^ zebrafish. **(A)** Image of the zebrafish wild type, *tfap2b* ^*+/−*^ and *tfap2* ^*−/−*^ intestine. Enteric neurons are shown in green and a white dash line delineates the intestine. **(B)** A significant reduced number of enteric neurons (*p < 0.0001, unpaired *t*-test) was found in the *tfap2b* ^*+/−*^ fish (n = 20) when compared to the wild type fish (n = 11). Neurons were counted and presented per 100 µm. Four out of 7 *tfap2b* ^*−/−*^ fish showed total aganglionosis. **(C)** Intestinal transit time was determined by the presence of a fluorescent pellet (red fluorescent) in the intestine 16 h after food intake (final measurement). To help evaluation, the zebrafish intestine was divided in 4 zones, proximal to distal, and empty. Only fish with fluorescent pellet in zone 1 at time 0 (initial measurement), were selected for this study. **(D)** A significant delay in intestinal transit time was observed in *tfap2b* ^*+/−*^ fish, as 75% of wild type fish had an empty intestine 16 h after food intake, in comparison with only 38.4% of *tfap2b* ^*+/−*^ fish (*p* < 0.0001, Proportion test). All *tfap2b* ^*−/−*^ fish had pellet retained in zone 2. **(E)** No difference in the ratio total body length to mouth-distal intestinal length was identified for wild type, tfap2b+/− and tfap2b−/− fish.

By counting the number of enteric neurons in 5 dpf F0 fish, we observed a reduction in neuronal numbers per 100 µm (mean = 24.431, SD ± 3.594) in crispants, when compared to uninjected or cas9-injected fish (mean = 30.144, SD ± 1.473; mean = 30.369, SD ± 1.864, respectively, *p* < 0.0001, Student’s *t* test; Supplementary Figure S2A). In addition, the length of the GI tract was evaluated in crispants and wild-type fish, by measuring the ratio of total body length to mouth-distal intestinal length. However, no significant difference was found at 5dpf (Supplementary Figure S2B). These results were consistently observed in F2 *tfap2b* ^*+/-*^ stable mutant fish (mean = 21.961, SD ± 4.087) when compared to wild type (mean = 30.296, SD ± 1.129, *p* < 0.0001, Student’s *t* test) as depicted in Figures 3A,B. Interestingly, we observed that the majority of *tfap2b* ^*−/−*^ showed total intestinal aganglionosis (4 out of 7).

To evaluate the effect of reduced enteric neuronal numbers on intestinal transit time, we performed an intestinal transit time assay in F2 fish. We divided the intestine in 4 zones (Figure 3C) and observed that while the majority of wild type fish showed an empty gut 16 h after feeding (75%), only 38.4% of *tfap2b* ^*+/−*^ showed the same (*p* < 0.0001, Proportion test). The majority of *tfap2b* ^*+/−*^ fish showed retention of the fluorescent pellet in proximal zones 2,3 and 4, suggesting a delay in intestinal transit when Tfap2b haploinsufficiency is detected. All the *tfap2b* ^*−/−*^ fish showed retention of pellet in zone 2 (Figure 3D). Furthermore, we evaluated the length of the GI tract in wild type, *tfap2b* ^*+/−*^, and *tfap2b* ^*−/−*^ by measuring the ratio of total body length to mouth-distal intestinal length. We found no difference between the groups (Figure 3E).

### Loss of Tfap2b Leads to a Reduction of *ednrbb* Transcript Levels

**FIGURE 4:**
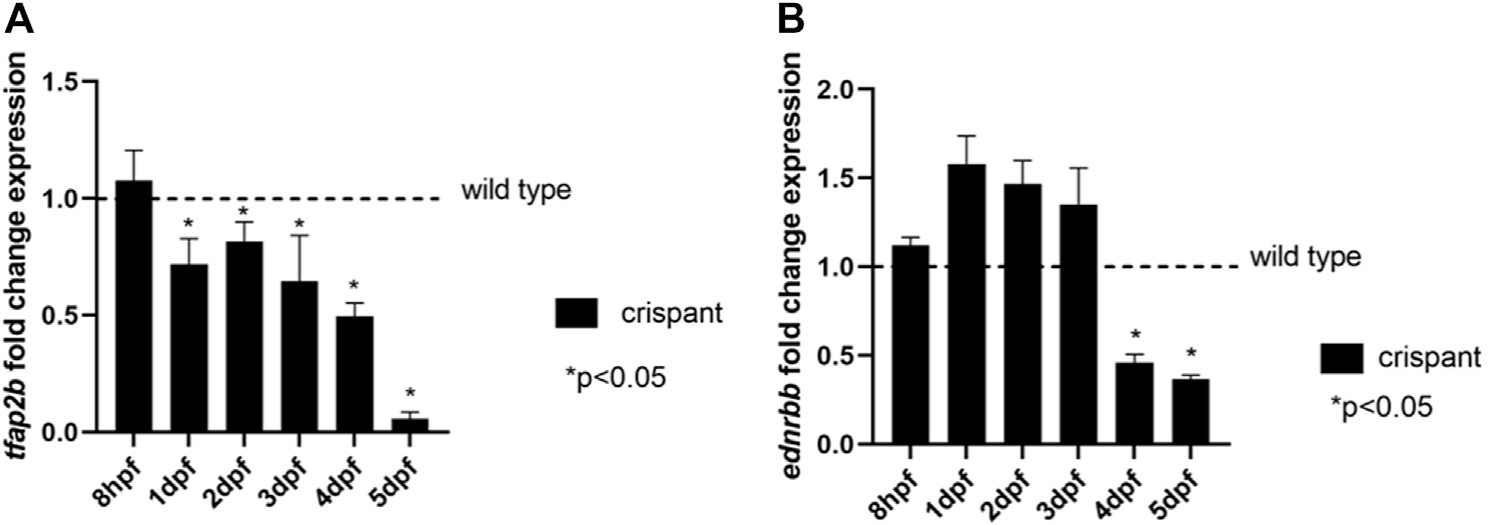
Expression levels of *tfap2b* and *ednrbb* during zebrafish development. **(A)** Expression levels of *tfap2b* in crispant fish at 8 hpf, 1, 2, 3, 4, and 5 dpf show a significant decrease when compared to wild type, starting from 1 dpf onwards (*p* < 0.05, unpaired *t*-test). **(B)** Expression levels of *ednrbb* in *tfap2b* crispants show a significant decrease when compared to wild type fish, at 4 and 5 dpf (*p* < 0.05, unpaired *t*-test).

TFAP2B was recently suggested to be one of transcription factors involved in the regulation of *EDNRB* expression (Ling and Sauka-Spengler, 2019). Since EDNRB is known to cause HSCR and is therefore, involved in GI development and function (Bondurand et al., 2018), we evaluated expression levels of this gene in our crispant *tfap2b* zebrafish model The following primers: 5′ GAT​CAC​TGA​GGG​AAA​AGC​TGG 3’ (forward) and 5′ AGT​TCG​TTT​GGA​TCA​GTG​TGC 3’ (reverse) were used to capture *tfap2b* transcript. Two *ednrb* orthologs are present in zebrafish, *ednrba* and *ednrbb*. However, only *ednrbb* seems to have an effect on GI development, as knocking down this gene in zebrafish showed less enteric neurons (Tilghman et al., 2019). Therefore, we decided to specifically determine the levels of *ednrbb* (5′ CCG​GTG​CGA​ATC​AAA​GAC​G 3’ (forward primer) and 5′ ACT​GCC​GAT​CAC​AAT​GTT​GG 3’ (reverse primer)) in our crispant fish at different time points of development (8 hpf to 5 dpf). As expected, a decrease in *tfap2b* transcript levels was detected at each time point analyzed starting from 1 dpf, when compared to wild type fish (*p* < 0.05, unpaired *t*-test; Figure 4A). A significant reduction in *ednrbb* transcript levels was also detected in crispants, but only at 4 and 5 dpf (*p* < 0.05, unpaired *t*-test; Figure 4B).

### Enteric Neuronal Differentiation is Not Affected in *tfap2b* ^*+/−*^ Fish

**FIGURE 5:**
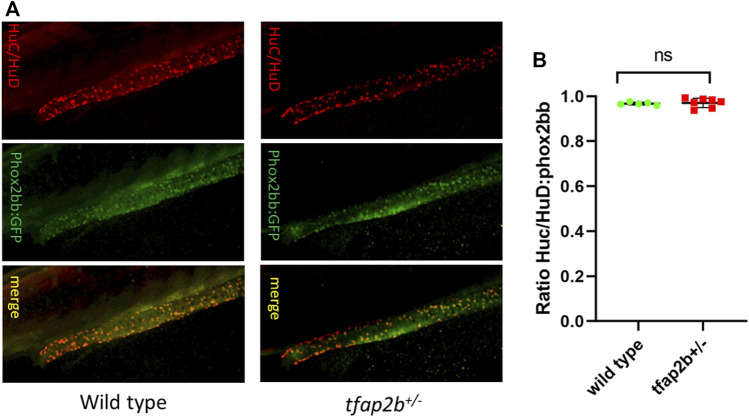
Differentiated enteric neurons in *tfap2b* ^*+/−*^ fish. **(A)** Confocal microscope images of HuC/HuD and phox2bb:GFP positive cells in wild type and *tfap2b* ^*+/−*^ F2 fish. **(B)** No significant difference in the ratio of HuC/HuD and phox2bb:GFP positive cells in wild type and *tfap2b* ^*+/−*^ F2 fish was found (*p* > 0.05, unpaired *t*-test).

Since EDNRB is involved in enteric neuronal differentiation, we evaluated if this process was affected in our *tfap2b* ^*+/−*^ fish. For this, a HuC/HuD immunostaining was performed to identify terminally differentiated enteric neurons, and HuC/HuD/phox2bb:GFP ratio was determined. Interestingly, we found no difference in the proportion of differentiated enteric neurons in *tfap2b* ^*+/−*^ (mean = 0.970, SD ± 0.020, n = 7) when compared to the wild type (mean = 0.967, SD ± 0.006, n = 5) (Figures 5A,B).

## Discussion

In this manuscript, we show that *TFAP2B* loss of function is associated to a congenital GI disorder, by identifying a *de novo* heterozygous deletion in this gene (c.602-5_606delTCTAGTTCCA), in a PIPO patient. Our *in vitro* studies showed that this deletion leads to exon 4 skipping and to the appearance of an early stop codon at position c.863. Consequently, a reduction in *TFAP2B* expression levels was observed. Although it is tempting to speculate that this reduction possibly occurred through the process of nonsense mediated decay, as we used the C-terminal Myc-tag to detect expression of TFAP2B, we cannot exclude the possibility that a small TFAP2B protein is expressed, as exon 1-3 are still intact. However, since this small protein will miss the DNA binding domain and the dimerization domain (Figure 1F), it is unlikely to be functional (Wankhade et al., 2000). Our zebrafish model confirmed the involvement of *TFAP2B* in GI development and function, as *tfap2b* crispant and *tfap2b* ^*+/−*^ F2 fish showed a reduction of enteric neurons and delayed intestinal transit time, with no change in total body to mouth-distal intestine length ratio when compared to wild type. Therefore, we classified this *de novo* deletion as a pathogenic variant based on AMCG standards and guidelines for interpretation of sequence variants. Interestingly, three loss of function variants in *TFAP2B* have been reported in control cohorts in GnomAD. However, since GI complaints might not be recorded in medical charts and can be easily missed due to variation in symptoms severity, we cannot definitively exclude that these controls have no clinical GI features. Such phenomenon is not uncommon in GI diseases, as mildest symptoms can be found in family members carrying the same pathogenic variant as the affected proband (Sribudiani et al., 2018).

TFAP2B together with TFAP2A, TFAP2C, TFAP2D and TFAP2E compose the AP-2 transcription factors family, known to form homo- or hetero-dimers with themselves, to function and bind specific DNA sequences as their targets (Hilger-Eversheim et al., 2000). These transcription factors are thought to stimulate proliferation and suppress terminal differentiation of specific cell types during embryonic development (Eckert et al., 2005), and were recently shown to be core regulators in orchestrating delineation of vagal neural crest cells, in an avian model (Ling and Sauka-Spengler, 2019). One of the genes found to be indirectly controlled by the TFAP2 family is *EDNRB*, a known gene required for enteric nervous system (ENS) development. In fact, mutations in *EDNRB* have been found in patients with HSCR, the most common enteric neuropathy characterized by lack of enteric neurons in the distal colon (Bondurand et al., 2018). Due to this link, we decided to evaluate transcript levels of *ednrbb* in our crispant *tfap2b* fish and observed a significant reduction of this gene, at 4 and 5dpf. Physiologically, at these developmental time points, colonization of the zebrafish gut by enteric neuronal progenitors is completed and neuronal differentiation is prominent (Howard et al., 2021; Kuil et al., 2021). Therefore, it was tempting to hypothesize that neuronal differentiation would be affected in the absence of *TFAP2B,* due to reduction of *ednrbb* levels. Our results showed that this was not the case (Figure 5B), as no difference in the ratio of HuC/D/phox2bb neurons was detected. EDN3/EDNRB signaling has also been reported to control proliferation of the enteric neural progenitors (Barlow et al., 2003; Nagy and Goldstein, 2006). Considering that our *tfap2b* ^*−/−*^ stable line show a HSCR phenotype, we suspect that a reduction in the number of progenitors is responsible for the reduction in enteric neurons identified.

Although this is the first time *TFAP2B* is linked to GI development, this is not the first time that this gene is involved in a congenital genetic disorder. Char syndrome and syndromic craniosynostosis are both caused by pathogenic mutations in this gene. However, while the majority of Char syndrome patients carry an autosomal dominant heterozygous missense variant in *TFAP2B*, nonsense, splice site, and missense variants have been identified in syndromic craniosynostosis (Figure 1E). These findings indicate a phenotypic variability resulting from *TFAP2B* defects, suggesting that variants in this gene lead to pleiotropic effects that vary in severity. Such phenomenon is not new, as variants in other genes are known to result in a spectrum of genetic disorders. Heterozygous Inactive Lysine Methyltransferase 2E (*KMT2E*) variants result in a spectrum of neurodevelopmental disorders, epilepsy, and functional GI disturbances (O’Donnell-Luria et al., 2019). Furthermore, variants in Coiled-Coil and C2 Domain Containing 2A (*CC2D2A*) have been recognized to cause a group of genetic disorders classified as ciliopathies. These include Meckel syndrome type 6, Joubert syndrome type 9, Bardet-Biedl syndrome and nephronophthisis (Lewis et al., 2019). Since we have now linked *TFAP2B* variants to PIPO, it will be interesting to evaluate if GI complaints are also present when assessing other obvious malformations, such as the ones described for Char syndrome and craniosynostosis. To date, there are no reports describing co-occurrence of Char syndrome and PIPO or craniosynostosis and PIPO, but this might be because GI symptoms were missed or considered to be secondary. Therefore, although it is too early to present *TFAP2B* as a new CIPO gene since only one patient has been identified, our results suggest that a better characterization of Char syndrome and syndromic craniosynostosis patients is needed, to improve counselling and define optimal treatment strategies.

## Data Availability

The datasets presented in this study can be found in online repositories. The names of the repository/repositories and accession number(s) can be found below: ClinVar database, accession number SCV002507296.

## References

[B1] Barlow A. De Graaff E. Pachnis V. (2003). Enteric Nervous System Progenitors Are Coordinately Controlled by the G Protein-Coupled Receptor EDNRB and the Receptor Tyrosine Kinase RET. Neuron 40, 905–916. 10.1016/S0896-6273(03)00730-X 14659090

[B2] Bondurand N. Dufour S. Pingault V. (2018). News from the Endothelin-3/EDNRB Signaling Pathway: Role during Enteric Nervous System Development and Involvement in Neural Crest-Associated Disorders. Dev. Biol. 444, S156–S169. 10.1016/j.ydbio.2018.08.014 30171849

[B3] Bonora E. Bianco F. Cordeddu L. Bamshad M. Francescatto L. Dowless D. (2015). Mutations in RAD21 Disrupt Regulation of Apob in Patients with Chronic Intestinal Pseudo-obstruction. Gastroenterology 148, 771–782. e11. 10.1053/j.gastro.2014.12.034 25575569 PMC4375026

[B4] Bradford Y. M. Van Slyke C. E. Ruzicka L. Singer A. Eagle A. Fashena D. (2022). Zebrafish Information Network, the Knowledgebase for *Danio rerio* Research. Genetics 220, iyac016. 10.1093/genetics/iyac016 35166825 PMC8982015

[B5] Brinkman E. K. Chen T. Amendola M. van Steensel B. (2014). Easy Quantitative Assessment of Genome Editing by Sequence Trace Decomposition. Nucleic Acids Res. 42, e168. 10.1093/nar/gku936 25300484 PMC4267669

[B6] Chambers B. E. Gerlach G. F. Clark E. G. Chen K. H. Levesque A. E. Leshchiner I. (2019). Tfap2a Is a Novel Gatekeeper of Nephron Differentiation during Kidney Development. Dev 146, dev172387. 10.1242/dev.172387 PMC6633607 31160420

[B7] Chen Y.-W. Zhao W. Zhang Z.-F. Fu Q. Shen J. Zhang Z. (2011). Familial Nonsyndromic Patent Ductus Arteriosus Caused by Mutations in TFAP2B. Pediatr. Cardiol. 32, 958–965. 10.1007/s00246-011-0024-7 21643846

[B8] Chetaille P. Preuss C. Preuss C. Burkhard S. Côté J.-M. Houde C. (2014). Mutations in SGOL1 Cause a Novel Cohesinopathy Affecting Heart and Gut Rhythm. Nat. Genet. 46, 1245–1249. 10.1038/ng.3113 25282101

[B9] Di Nardo G. Di Lorenzo C. Lauro A. Stanghellini V. Thapar N. Karunaratne T. B. (2017). Chronic Intestinal Pseudo-obstruction in Children and Adults: Diagnosis and Therapeutic Options. Neurogastroenterol. Motil. 29, e12945. 10.1111/nmo.12945 27683196

[B10] Downes T. J. Cheruvu M. S. Karunaratne T. B. De Giorgio R. Farmer A. D. (2018). Pathophysiology, Diagnosis, and Management of Chronic Intestinal Pseudo-obstruction. J. Clin. Gastroenterol. 52, 477–489. 10.1097/MCG.0000000000001047 29877952

[B11] Eckert D. Buhl S. Weber S. Jäger R. Schorle H. (2005). The AP-2 Family of Transcription Factors. Genome Biol. 6, 246. 10.1186/gb-2005-6-13-246 16420676 PMC1414101

[B12] Edward H. L. D'Gama A. M. Wojcik M. H. Brownstein C. A. Kenna M. A. Grant P. E. (2019). A Novel Missense Mutation inTFAP2Bassociated with Char Syndrome and Central Diabetes Insipidus. Am. J. Med. Genet. 179, 1299–1303. 10.1002/ajmg.a.61150 31012281

[B13] Gamboa H. E. Sood M. (2019). Pediatric Intestinal Pseudo-obstruction in the Era of Genetic Sequencing. Curr. Gastroenterol. Rep. 21, 70. 10.1007/s11894-019-0737-y 31848803

[B14] Gargiulo A. Auricchio R. Barone M. V. Cotugno G. Reardon W. Milla P. J. (2007). Filamin A Is Mutated in X-Linked Chronic Idiopathic Intestinal Pseudo-obstruction with Central Nervous System Involvement. Am. J. Hum. Genet. 80, 751–758. 10.1086/513321 17357080 PMC1852717

[B15] Giordano C. Powell H. Leopizzi M. De Curtis M. Travaglini C. Sebastiani M. (2009). Fatal Congenital Myopathy and Gastrointestinal Pseudo-obstruction Due to POLG1 Mutations. Neurology 72, 1103–1105. 10.1212/01.wnl.0000345002.47396.e1 19307547 PMC2821839

[B16] Halim D. Hofstra R. M. W. Signorile L. Verdijk R. M. Van Der Werf C. S. Sribudiani Y. (2016). ACTG2variants Impair Actin Polymerization in Sporadic Megacystis Microcolon Intestinal Hypoperistalsis Syndrome. Hum. Mol. Genet. 25, 571–583. 10.1093/hmg/ddv497 26647307

[B17] Hilger-Eversheim K. Moser M. Schorle H. Buettner R. (2000). Regulatory Roles of AP-2 Transcription Factors in Vertebrate Development, Apoptosis and Cell-Cycle Control. Gene 260, 1–12. 10.1016/S0378-1119(00)00454-6 11137286

[B18] Hong S. J. Huh Y. H. Leung A. Choi H. J. Ding Y. Kang U. J. (2011). Transcription Factor AP-2β Regulates the Neurotransmitter Phenotype and Maturation of Chromaffin Cells. Mol. Cell. Neurosci. 46, 245–251. 10.1016/j.mcn.2010.09.007 20875861 PMC3139976

[B19] Hong S. J. Lardaro T. Oh M. S. Huh Y. Ding Y. Kang U. J. (2008). Regulation of the Noradrenaline Neurotransmitter Phenotype by the Transcription Factor AP-2β. J. Biol. Chem. 283, 16860–16867. 10.1074/jbc.M709106200 18424435 PMC2423267

[B20] Howard A. G. Baker P. A. Ibarra-García-Padilla R. Moore J. A. Rivas L. J. Tallman J. J. (2021). An Atlas of Neural Crest Lineages along the Posterior Developing Zebrafish at Single-Cell Resolution. Elife 10, 1–31. 10.7554/eLife.60005 PMC7886338 33591267

[B21] Jenkins Z. A. Macharg A. Chang C.-Y. van Kogelenberg M. Morgan T. Frentz S. (2018). Differential Regulation of twoFLNAtranscripts Explains Some of the Phenotypic Heterogeneity in the Loss-Of-Function Filaminopathies. Hum. Mutat. 39, 103–113. 10.1002/humu.23355 29024177

[B22] Ji W. Benson M. A. Bhattacharya S. Chen Y. Hu J. Li F. (2014). Characterization of Transcription Factor AP-2 Beta Mutations Involved in Familial Isolated Patent Ductus Arteriosus Suggests Haploinsufficiency. J. Surg. Res. 188, 466–472. 10.1016/j.jss.2014.01.015 24507797 PMC4594773

[B23] Jin K. Jiang H. Xiao D. Zou M. Zhu J. Xiang M. (2015). Tfap2a and 2b Act Downstream of Ptf1a to Promote Amacrine Cell Differentiation during Retinogenesis. Mol. Brain 8, 1–14. 10.1186/s13041-015-0118-x 25966682 PMC4429372

[B24] Khetyar M. Syrris P. Tinworth L. Abushaban L. Carter N. (2008). NovelTFAP2BMutation in Nonsyndromic Patent Ductus Arteriosus. Genet. Test. 12, 457–459. 10.1089/gte.2008.0015 18752453

[B25] Knowles C. H. De Giorgio R. Kapur R. P. Bruder E. Farrugia G. Geboes K. (2010). The London Classification of Gastrointestinal Neuromuscular Pathology: Report on Behalf of the Gastro 2009 International Working Group. Gut 59, 882–887. 10.1136/gut.2009.200444 20581236

[B26] Kraja A. T. Chasman D. I. North K. E. Reiner A. P. Yanek L. R. Kilpeläinen T. O. (2014). Pleiotropic Genes for Metabolic Syndrome and Inflammation. Mol. Genet. Metab. 112, 317–338. 10.1016/j.ymgme.2014.04.007 24981077 PMC4122618

[B27] Kuil L. E. Chauhan R. K. Cheng W. W. Hofstra R. M. W. Alves M. M. (2021). Zebrafish: A Model Organism for Studying Enteric Nervous System Development and Disease. Front. Cell. Dev. Biol. 8, 1–15. 10.3389/fcell.2020.629073 PMC7859111 33553169

[B28] Kuil L. E. Oosterhof N. Geurts S. N. Van Der Linde H. C. Meijering E. Van Ham T. J. (2019). Reverse Genetic Screen Reveals that Il34 Facilitates Yolk Sac Macrophage Distribution and Seeding of the Brain. DMM Dis. Model. Mech. 12, 1–12. 10.1242/dmm.037762 PMC6451432 30765415

[B29] Lewis W. R. Bales K. L. Revell D. Z. Croyle M. J. Engle S. E. Song C. J. (2019). Mks6 Mutations Reveal Tissue‐ and Cell Type‐specific Roles for the Cilia Transition Zone. FASEB J. 33, 1440–1455. 10.1096/fj.201801149R 30133325 PMC6355093

[B30] Li H. Durbin R. (2009). Fast and Accurate Short Read Alignment with Burrows-Wheeler Transform. Bioinformatics 25, 1754–1760. 10.1093/bioinformatics/btp324 19451168 PMC2705234

[B31] Ling I. T. C. Sauka-Spengler T. (2019). Early Chromatin Shaping Predetermines Multipotent Vagal Neural Crest into Neural, Neuronal and Mesenchymal Lineages. Nat. Cell. Biol. 21, 1504–1517. 10.1038/s41556-019-0428-9 31792380 PMC7188519

[B32] Mani A. Radhakrishnan J. Farhi A. Carew K. S. Warnes C. A. Nelson-Williams C. (2005). Syndromic Patent Ductus Arteriosus: Evidence for Haploinsufficient TFAP2B Mutations and Identification of a Linked Sleep Disorder. Proc. Natl. Acad. Sci. U.S.A. 102, 2975–2979. 10.1073/pnas.0409852102 15684060 PMC549488

[B33] Milunsky A. Baldwin C. Zhang X. Primack D. Curnow A. Milunsky J. (2017). Diagnosis of Chronic Intestinal Pseudo-obstruction and Megacystis by Sequencing the ACTG2 Gene. J. Pediatr. Gastroenterol. Nutr. 65, 384–387. 10.1097/MPG.0000000000001608 28422808 PMC5610062

[B34] Moser M. Dahmen S. Kluge R. Gröne H. Dahmen J. Kunz D. (2003). Terminal Renal Failure in Mice Lacking Transcription Factor AP-2β. Lab. Invest. 83, 571–578. 10.1097/01.LAB.0000064703.92382.50 12695560

[B35] Muto M. Matsufuji H. Tomomasa T. Nakajima A. Kawahara H. Ida S. (2014). Pediatric Chronic Intestinal Pseudo-obstruction Is a Rare, Serious, and Intractable Disease: A Report of a Nationwide Survey in Japan. J. Pediatr. Surg. 49, 1799–1803. 10.1016/j.jpedsurg.2014.09.025 25487487

[B36] Nagy N. Goldstein A. M. (2006). Endothelin-3 Regulates Neural Crest Cell Proliferation and Differentiation in the Hindgut Enteric Nervous System. Dev. Biol. 293, 203–217. 10.1016/j.ydbio.2006.01.032 16519884

[B37] Nechiporuk A. Linbo T. Poss K. D. Raible D. W. (2007). Specification of Epibranchial Placodes in Zebrafish. Development 134, 611–623. 10.1242/dev.02749 17215310

[B38] Nishino I. Spinazzola A. Hirano M. (1999). Thymidine Phosphorylase Gene Mutations in MNGIE, a Human Mitochondrial Disorder. Science 283, 689–692. 10.1126/science.283.5402.689 9924029

[B39] O'Donnell-Luria A. H. Pais L. S. Faundes V. Wood J. C. Sveden A. Luria V. (2019). Heterozygous Variants in KMT2E Cause a Spectrum of Neurodevelopmental Disorders and Epilepsy. Am. J. Hum. Genet. 104, 1210–1222. 10.1016/j.ajhg.2019.03.021 31079897 PMC6556837

[B40] Prasad M. S. Charney R. M. García‐Castro M. I. (2019). Specification and Formation of the Neural Crest: Perspectives on Lineage Segregation. Genesis 57, e23276–21. 10.1002/dvg.23276 30576078 PMC6570420

[B41] Ravenscroft G. Pannell S. O'Grady G. Ong R. Ee H. C. Faiz F. (2018). Variants in ACTG2 Underlie a Substantial Number of Australasian Patients with Primary Chronic Intestinal Pseudo-obstruction. Neurogastroenterol. Motil. 30, e13371–9. 10.1111/nmo.13371 29781137

[B42] Richards S. Aziz N. Bale S. Bick D. Das S. Gastier-Foster J. (2015). Standards and Guidelines for the Interpretation of Sequence Variants: A Joint Consensus Recommendation of the American College of Medical Genetics and Genomics and the Association for Molecular Pathology. Genet. Med. 17, 405–424. 10.1038/gim.2015.30 25741868 PMC4544753

[B43] Satoda M. Zhao F. Diaz G. A. Burn J. Goodship J. Davidson H. R. (2000). Mutations in TFAP2B Cause Char Syndrome, a Familial Form of Patent Ductus Arteriosus. Nat. Genet. 25, 42–46. 10.1038/75578 10802654

[B44] Schmidt M. Huber L. Majdazari A. Schütz G. Williams T. Rohrer H. (2011). The Transcription Factors AP-2β and AP-2α Are Required for Survival of Sympathetic Progenitors and Differentiated Sympathetic Neurons. Dev. Biol. 355, 89–100. 10.1016/j.ydbio.2011.04.011 21539825

[B45] Sribudiani Y. Chauhan R. K. Alves M. M. Petrova L. Brosens E. Harrison C. (2018). Identification of Variants in RET and IHH Pathway Members in a Large Family with History of Hirschsprung Disease. Gastroenterology 155, 118–129. e6. 10.1053/j.gastro.2018.03.034 29601828

[B46] Tanasubsinn N. Sittiwangkul R. Pongprot Y. Kawasaki K. Ohazama A. Sastraruji T. (2017). TFAP2B Mutation and Dental Anomalies. J. Hum. Genet. 62, 769–775. 10.1038/jhg.2017.37 28381879 PMC5537417

[B47] Thapar N. Saliakellis E. Benninga M. A. Borrelli O. Curry J. Faure C. (2018). Paediatric Intestinal Pseudo-obstruction: Evidence and Consensus-Based Recommendations from an ESPGHAN-Led Expert Group. J. Pediatr. Gastroenterol. Nutr. 66, 991–1019. 10.1097/MPG.0000000000001982 29570554

[B48] Thisse B. Thisse C. (2004). Fast Release Clone: A High Throughput Expression Analysis. ZFIN Direct Data Submiss. AvaliableAt: http://zfin.org .

[B49] Tilghman J. M. Ling A. Y. Turner T. N. Sosa M. X. Krumm N. Chatterjee S. (2019). Molecular Genetic Anatomy and Risk Profile of Hirschsprung's Disease. N. Engl. J. Med. 380, 1421–1432. 10.1056/nejmoa1706594 30970187 PMC6596298

[B50] Timberlake A. T. Jin S. C. Nelson-Williams C. Wu R. Furey C. G. Islam B. (2019). Mutations in TFAP2B and Previously Unimplicated Genes of the BMP, Wnt, and Hedgehog Pathways in Syndromic Craniosynostosis. Proc. Natl. Acad. Sci. U. S. A. 116116, 15116–15121. 10.1073/pnas.1902010.1073/pnas.191289311610.1073/pnas.1902041116 PMC6660739 31292255

[B51] Uyttebroek L. Shepherd I. T. Harrisson F. Hubens G. Blust R. Timmermans J.-P. (2010). Neurochemical Coding of Enteric Neurons in Adult and Embryonic Zebrafish (*Danio rerio*). J. Comp. Neurol. 518, 4419–4438. 10.1002/cne.22464 20853514 PMC4014623

[B52] Van Der Werf C. S. Sribudiani Y. Verheij J. B. G. M. Carroll M. O’Loughlin E. Chen C.-H. (2013). Congenital Short Bowel Syndrome as the Presenting Symptom in Male Patients with FLNA Mutations. Genet. Med. 15, 310–313. 10.1038/gim.2012.123 23037936

[B54] Van Goethem G. Schwartz M. Löfgren A. Dermaut B. Van Broeckhoven C. Vissing J. (2003). Novel POLG Mutations in Progressive External Ophthalmoplegia Mimicking Mitochondrial Neurogastrointestinal Encephalomyopathy. Eur. J. Hum. Genet. 11, 547–549. 10.1038/sj.ejhg.5201002 12825077

[B55] Wankhade S. Yu Y. Weinberg J. Tainsky M. A. Kannan P. (2000). Characterization of the Activation Domains of AP-2 Family Transcription Factors. J. Biol. Chem. 275, 29701–29708. 10.1074/jbc.M000931200 10899156

[B56] Xiong F. Li Q. Zhang C. Chen Y. Li P. Wei X. (2013). Analyses of GATA4, NKX2.5, and TFAP2B Genes in Subjects from Southern China with Sporadic Congenital Heart Disease. Cardiovasc. Pathol. 22, 141–145. 10.1016/j.carpath.2012.07.001 22959235

[B57] Zhao F. Weismann C. G. Satoda M. Ella Pierpont M. M. Sweeney E. Thompson E. M. (2001). Novel TFAP2B Mutations that Cause Char Syndrome Provide a Genotype-Phenotype Correlation. Am. J. Hum. Genet. 69, 695–703. 10.1086/323410 11505339 PMC1226056

