# Supplementary material for: TFAP2B Haploinsufficiency Impacts Gastrointestinal Function and Leads to Pediatric Intestinal Pseudo-obstruction
Source: Front Cell Dev Biol. 2022 Jul 8;10:901824. doi: 10.3389/fcell.2022.901824 (PMC9304996; doi:10.3389/fcell.2022.901824)
Supplement: Supplementary file 1 [file DataSheet1.docx]

Supplementary Material

Supplementary Table 1. **List of site directed mutagenesis primers used in this study.**

| Gene | Sequence |
| --- | --- |
| TFAP2B-sdm-C706T-F | CGTCCCAGGCtGTTTGTCTCT |
| TFAP2B-sdm-C706T-R | GAGCAAAACACCTCGCCG |
| TFAP2B-sdm-C898T-F | CGCGGGCAGGtGCAAAGCAGC |
| TFAP2B-sdm-C898T-R | GGTAAATTCAAACCGATTTTTTCTAGCCTTTCTCGC |
| TFAP2B-sdm-delex4-F | AGCCAAATCGAAAAATGG |
| TFAP2B-sdm-delex4-R | CTTTTTTAATGACAGACTGG |
| TFAP2B-sdm-C1144T-F | AGGGAACAGCtGACCCAGCCC |
| TFAP2B-sdm-C1144T-R | ATCGGTGTCCGGTCCTGC |

Supplementary Figure 1a.


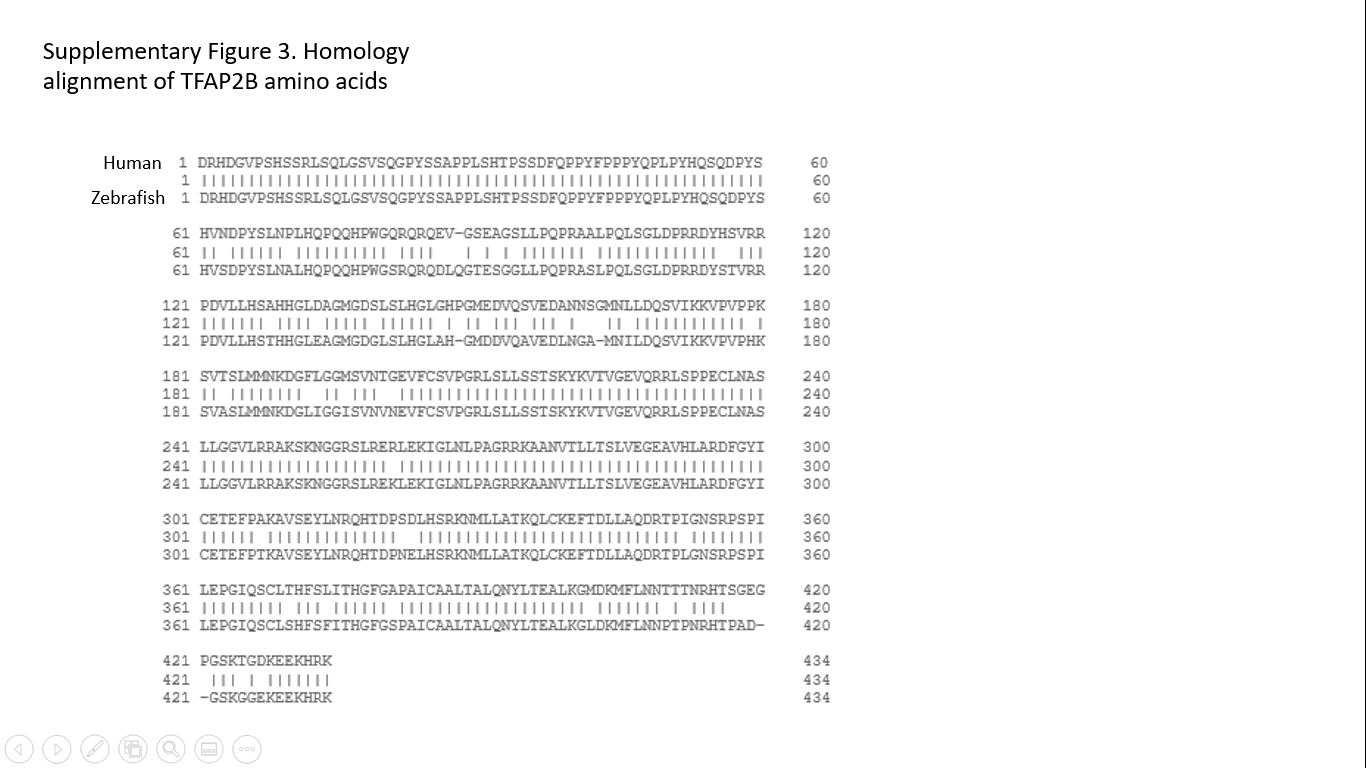


Supplementary Figure 1b.


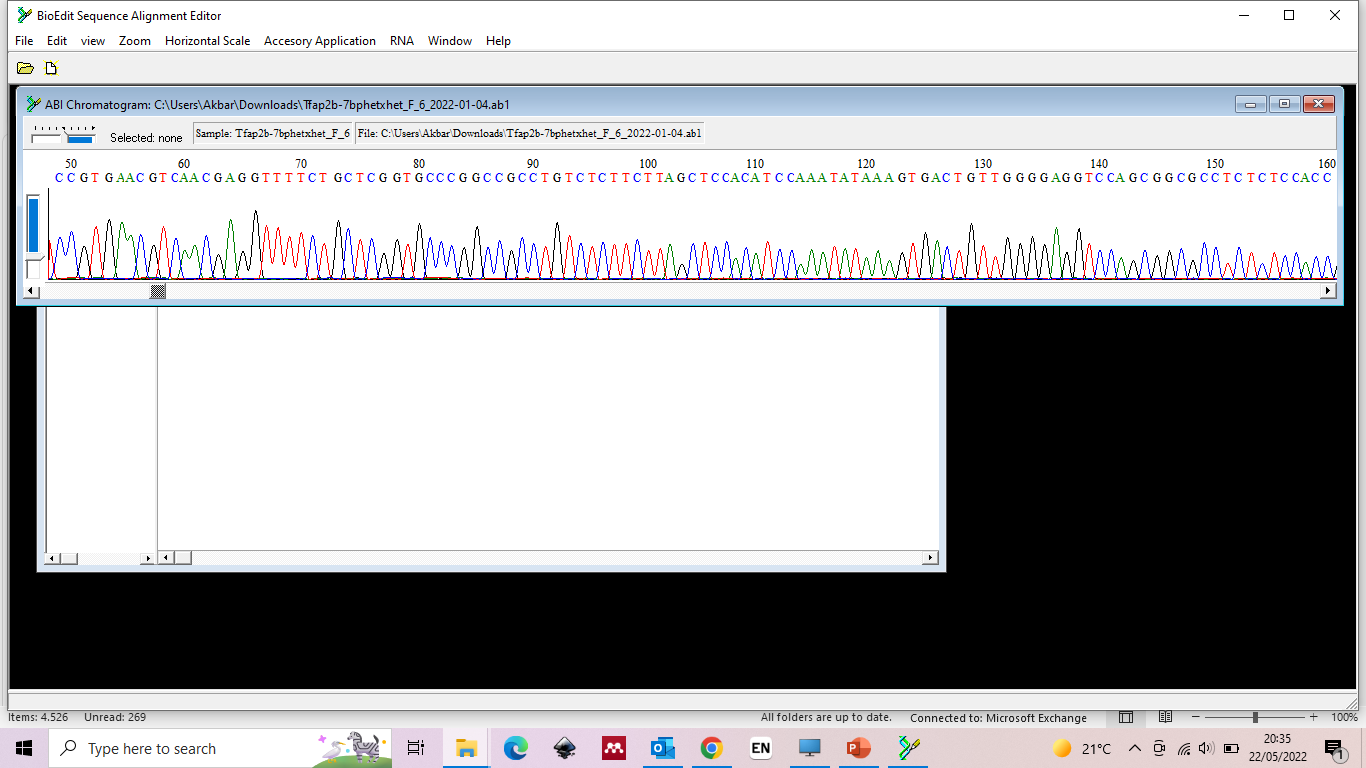

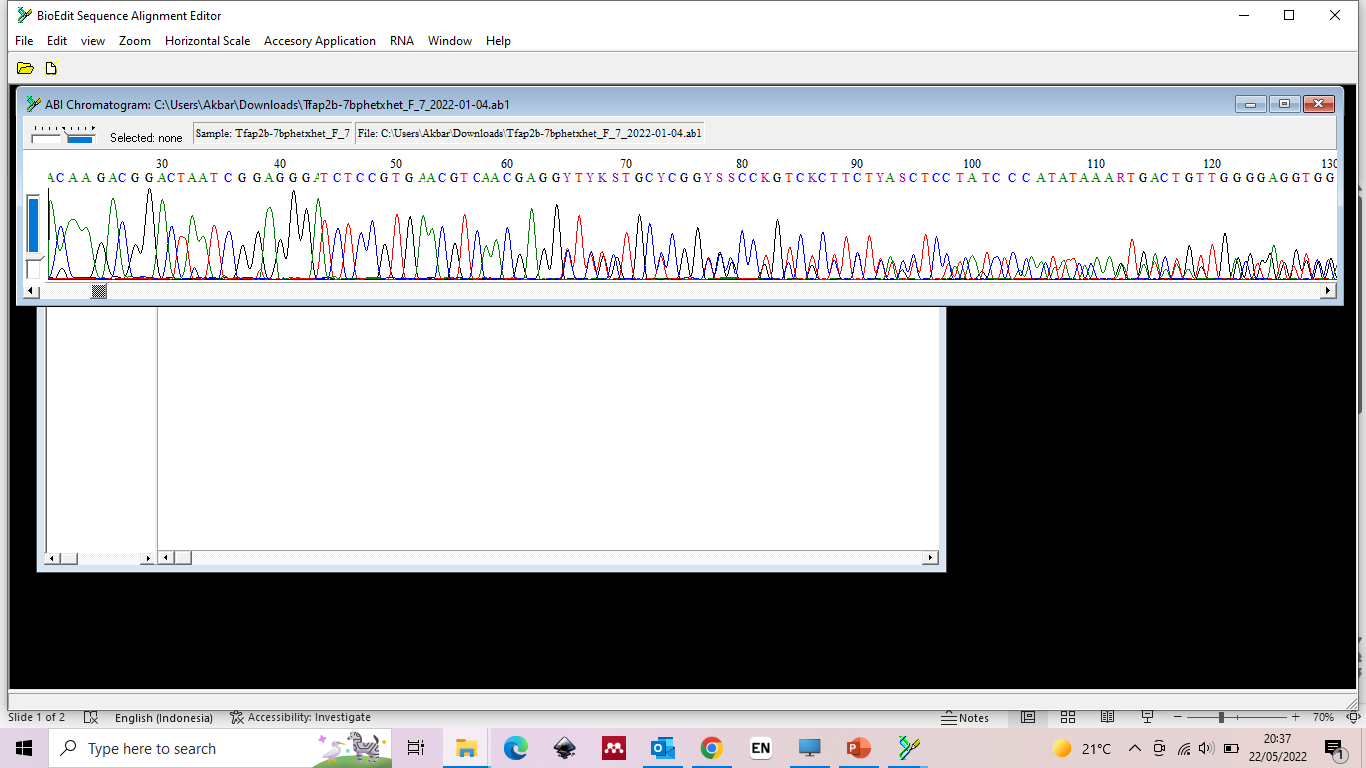


Wild type

F1 *tfap2b^+/-^*

Supplementary Figure 1c.


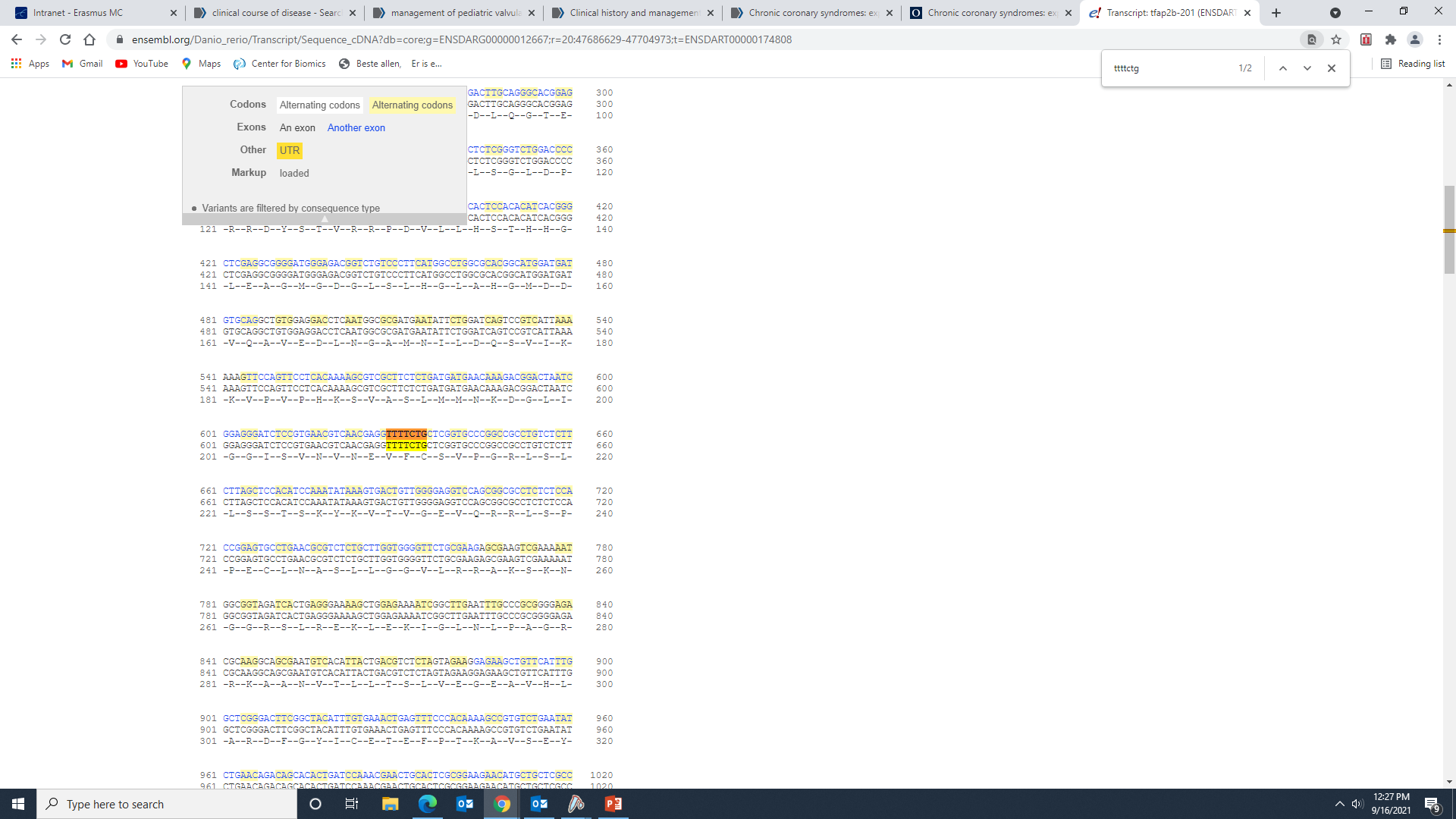


Stop codon

Deletion 7bp

Supplementary Figure 1. **Generation of a *tfap2b^+/-^* zebrafish line.** a). The alignment of the human (NM_003221.4) and zebrafish (ENSDART00000174808.2) TFAP2B protein shows 88.71% homology (BLAST Tools, ensemble.org). b). Sequencing validation of F1 *tfap2b*^+/-^ zebrafish generated from crispants (F0), shows the presence of a heterozygous deletion of 7 base pairs (bp) (c.629-635delTTTTCTG). c). The c.629-635delTTTTCTG leads to a frame shift and results in the appearance of a stop codon at position c.689.

Supplementary Figure 2a.


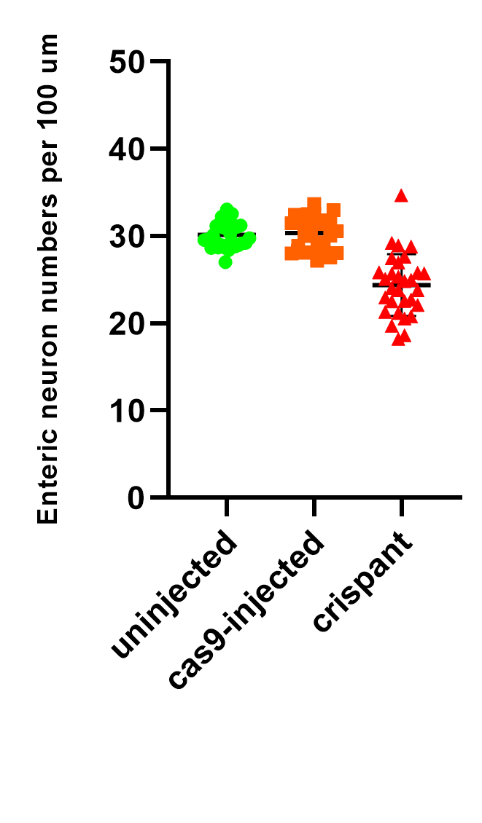


Supplementary Figure 2b.


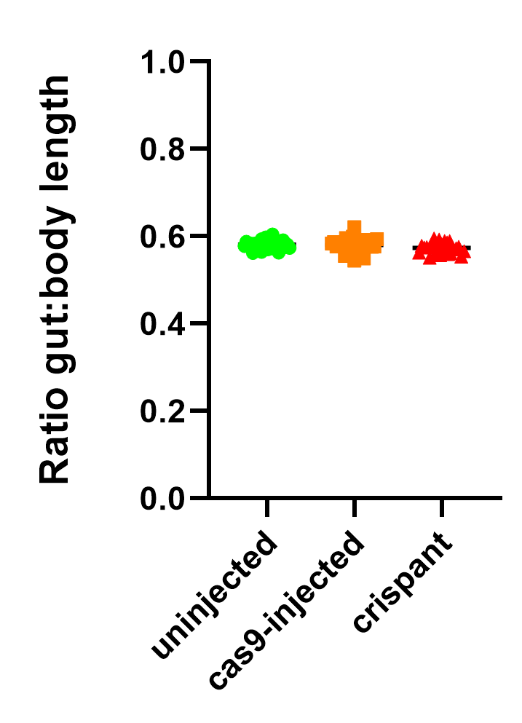


Supplementary Figure 2. **Effect of *tfap2b* reduction in the number of enteric neurons and in the gut vs total body length ratio**. a). A significant decreased number of enteric neurons (*p<0.0001, Student’s T test) was found in crispant fish (n=30, mean= 24.431, SD± 3.594), when compared to uninjected (n=30, mean= 30.144, SD±1.473) or cas9-injected fish (n=30, mean= 30.369, SD± 1.864). Neurons were counted and presented per 100µm. b). No effect on the gut and body length was detected in crispant fish when compared to uninjected or cas9-injected fish.
